# Investigating the role of genetic variation in *vgll3* and *six6* in the domestication of gilthead seabream (*Sparus aurata* Linnaeus) and European seabass (*Dicentrarchus labrax* Linnaeus)

**DOI:** 10.1002/ece3.10727

**Published:** 2023-11-16

**Authors:** Aristotelis Moulistanos, Theopisti Nikolaou, Smaragda Sismanoglou, Konstantinos Gkagkavouzis, Nikoleta Karaiskou, Efthimia Antonopoulou, Alexandros Triantafyllidis, Spiros Papakostas

**Affiliations:** ^1^ Department of Genetics, Development & Molecular Biology, School of Biology, Faculty of Science Aristotle University of Thessaloniki Thessaloniki Greece; ^2^ Genomics and Epigenomics Translational Research (GENeTres) Center for Interdisciplinary Research and Innovation (CIRI‐AUTH), Balkan Center Thessaloniki Greece; ^3^ Department of Zoology, School of Biology Aristotle University of Thessaloniki Thessaloniki Greece; ^4^ Department of Science and Technology International Hellenic University Thessaloniki Greece

**Keywords:** artificial selection, candidate gene approach, fish farming, genome scans, marine teleost

## Abstract

Gene function conservation is crucial in molecular ecology, especially for key traits like growth and maturation in teleost fish. The *vgll3* and *six6* genes are known to influence age‐at‐maturity in Atlantic salmon, but their impact on other fish species is poorly understood. Here, we investigated the association of *vgll3* and *six6* in the domestication of gilthead seabream and European seabass, both undergoing selective breeding for growth‐related traits in the Mediterranean. We analysed two different sets of samples using two different genotyping approaches. The first dataset comprised farmed and wild populations from Greece, genotyped for SNPs within the two genes (‘gene‐level genotyping’). The second dataset examined 300–600 k SNPs located in the chromosomes of the two genes, derived from a meta‐analysis of a Pool‐Seq experiment involving farmed and wild populations distributed widely across the Mediterranean (‘chromosome‐level genotyping’). The gene‐level analysis revealed a statistically significant allele frequency differences between farmed and wild populations on both genes in each species. This finding was partially supported by the chromosome‐level analysis, identifying highly differentiated regions may be involved in the domestication process at varying distances from the candidate genes. Noteworthy genomic features were found, such as a CpG island in gilthead seabream and novel candidate genes in European seabass, warranting further investigation. These findings support a putative role of *vgll3* and *six6* in the maturation and growth of gilthead seabream and European seabass, emphasizing the need for further research on their conserved function.

## Graphical abstract

The research investigates the impact of *vgll3* and *six6* genes, known to influence age‐at‐maturity in Atlantic salmon, in gilthead seabream and European seabass, both undergoing selective breeding for growth‐related traits in the Mediterranean. Two distinct genotyping approaches, gene‐level and chromosome‐level, were employed, revealing significant domestication effects on both genes in each species, with noteworthy genomic features identified. These findings indirectly support a potential role of *vgll3* and *six6* in the maturation and growth of gilthead seabream and European seabass, highlighting the need for further research on their conserved function.

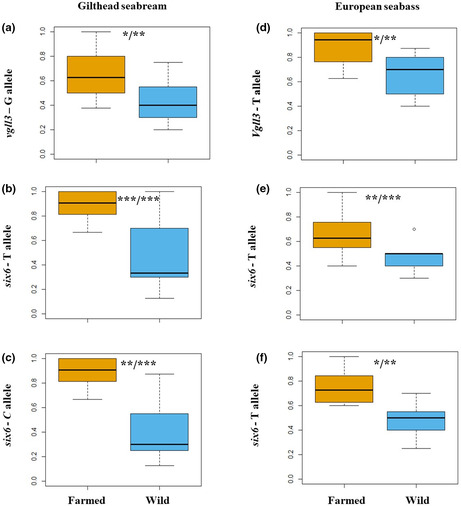

## 1 INTRODUCTION

The evolutionary conservation of gene function is a valuable hypothesis for investigating candidate genes that regulate similar traits across different species. By leveraging the accumulated knowledge and insights gained from often costly and labour‐intensive genetic and ecological experiments conducted in model species, researchers can apply these insights to other ecologically or commercially important species. This strategy is widely used in molecular ecology within the framework of the candidate gene approach, to address diverse research questions, most notably the genetic basis of various traits in a broad range of species (e.g. Brown et al., 2013; Hemmer‐Hansen et al., 2011; Mueller et al., 2013; Wilkie et al., 2017). Assessing evolutionary conservation of function has also significant evolutionary implications. By examining the degree of conservation of genomic architectures across phylogenies, researchers can gain insights into the rates of evolutionary change, the relative importance of biological functions and the origins of evolutionary novelty (e.g. Kadereit et al., 2008; Lieberman, 2006; Martínez Corrales & Alic, 2020; Struemph & Henderson, 2021; Xiao et al., 2015).

Growth and maturation are essential life‐history traits that significantly impact the physiology and fitness of teleost fish. However, the genetic basis of these traits remains poorly understood in several fish species (Boglione et al., 2013; Mobley et al., 2021) largely because they are polygenic and highly influenced by the environment (Mobley et al., 2021; Mohamed et al., 2019; Sinclair‐Waters et al., 2020). Nonetheless, both traits are economically important with relatively high heritability (Gong et al., 2022; Navarro et al., 2009; Wang et al., 2015), rendering them suitable for selective breeding as they can be easily measured and targeted in breeding programs (Chavanne et al., 2016; Yue, 2014). In some fish species, evidence suggests an antagonistic relationship between growth and maturation. For example, individuals with delayed maturation exhibit earlier somatic growth, making them preferred candidates for selective breeding in aquaculture (Sinclair‐Waters et al., 2020; Yue, 2014). Despite extensive molecular efforts to understand the genomic architecture underlying these traits, incongruent findings suggest that growth and maturation in teleosts may not share large‐effect genes or similar regulatory checkpoints (Ali et al., 2020; Gong et al., 2022; Wang et al., 2015; Yassumoto et al., 2020; Zhou et al., 2019).

Intriguingly, recent studies have shed light on the molecular basis of maturation in Atlantic salmon (*Salmo salar* Linnaeus), revealing evidence of functional conservation across large evolutionary distances. The vestigial‐like family member 3 (*vgll3*) and SIX homeobox 6 (*six6*) genes have emerged as strong predictors of age‐at‐maturity in both wild and farmed Atlantic salmon selected for growth (Ayllon et al., 2015; Barson et al., 2015; Czorlich et al., 2022; Sinclair‐Waters et al., 2020). The *vgll3* was further shown to impact the body condition defined as deviation from the slope of logarithmic mass on logarithmic length, of the species (Debes et al., 2021). The expression profile of *six6* has been linked to the evolutionary conserved Hippo signalling pathway (Kurko et al., 2020), and has also been identified as a maturation candidate gene in other salmonid species, and in particular, in Sockeye salmon (*Oncorhynchus nerka* Walbaum) and Steelhead trout (*Oncorhynchus mykiss* Walbaum), but not in Chinook salmon (*Oncorhynchus tshawytscha* Walbaum) and Coho salmon (*Oncorhynchus kisutch* Walbaum) (Waters et al., 2021). In non‐salmonid species like zebrafish (*Danio rerio* Hamilton), *vgll3* has been suggested to play a role in growth during early development (Pennonen, 2017). Moreover, both genes have been associated with maturation and growth‐related traits in mammals, specifically age‐at‐menarche and pubertal height growth in humans (Cousminer et al., 2013; Perry et al., 2014) as well as puberty in beef cattle (Cánovas et al., 2014). Given this evidence, an open question remains whether *vgll3* and *six6* regulate growth and/or maturation in other teleost fish, especially in those of commercial value.

To this end, the gilthead seabream (*Sparus aurata*) and the European seabass (*Dicentrarchus labrax*) are economically important fish species in the Mediterranean region, with unresolved genetic bases of maturation and somatic growth (Loukovitis et al., 2011, 2012; Louro et al., 2016; Massault et al., 2010). The gilthead seabream is a protandrous hermaphrodite fish species, initially developing as male (maturing during the first or second year of age) and transitioning to female after about 2 years. Within the Sparidae family, social control mechanisms likely maintain a balanced sex ratio, as not all individuals undergo this process (Brown, 2003). The European seabass is an amphigonic sexually dimorphic organism, where sex is influenced by both genetic and environmental factors and the first sexual maturity occurs between 2 and 4 years of age in Mediterranean Sea. Elevated temperatures used to promote faster growth in hatcheries often result in male‐biased farmed populations (Vandeputte et al., 2019). Both species have been the focus of selective breeding programs for over a decade, targeting traits such as growth, food conversion efficiency and disease resistance, with the aim of improving their culturing characteristics and profitability (Chavanne et al., 2016). This adaptation process of fish to captivity is known as fish domestication (Teletchea, 2021), and within this context, it is closely associated with the growth and maturation traits for which the *vgll3* and *six6* genes are considered strong candidates.

In this study, we aimed to investigate the relationship between genetic variation in the *vgll3* and *six6* genes and the process of domestication in gilthead seabream and European seabass. To ensure the reliability of our findings, we genotyped two distinct sample collections. First, we employed Sanger sequencing to genotype polymorphic SNPs within the two candidate genes for each targeted species. Specifically, we genotyped 91 samples for *vgll3* and 79 samples for *six6* of gilthead seabream. For European seabass, we genotyped 91 samples for *vgll3* and 87 samples for *six6*. This genotyping approach is consistently referred to as ‘gene‐level genotyping’ throughout the study. Second, we analysed the Illumina Pool‐Seq data produced by Peñaloza et al. (2021), which included 14 farmed and 10 wild populations of gilthead seabream, as well as 12 farmed and 12 wild populations of European seabass across the Mediterranean region. Peñaloza et al. (2021) utilized this dataset to develop a SNP chip for population genomic analyses in the two species. In our study, we used this dataset to explore the chromosome‐wide impact of domestication on the genetic variation of the chromosomes containing the two candidate genes in each species. This approach is consistently referred to as ‘chromosome‐level genotyping’ throughout the study. By integrating the two sets of genotypes, we examined whether there was a correlation between the process of domestication in the two fish species and the detected patterns of genetic variation in *vgll3* and *six6* and their surrounding regions. Subsequent studies planned may extend our conclusions encompassing genome‐wide effects.

## 2 MATERIALS AND METHODS

### 2.1 Gene‐level genotyping

We employed Sanger sequencing to genotype polymorphic SNPs in the gene regions of *vgll3* and *six6* in the studied samples. Subsequently, we statistically analysed the differences in allele frequencies between farmed and wild populations to assess the effect of the domestication process.

#### 2.1.1 Sample collection and DNA extractions

We sampled nine farmed and 11 wild gilthead seabream and seven farmed and 11 wild European seabass populations (Table 1). Genomic DNA was extracted using the protocol by Hillis et al. (1996). No phenotypic measurements were taken.

**TABLE 1:** Information on the samples used for gene‐level genotyping of gilthead seabream and European seabass populations.

Gilthead seabream (*Sparus aurata*)					European seabass (*Dicentrarchus labrax*)				
Pop_ID	Origin (locality)	Year	No of individuals		Pop_ID	Origin (locality)	Year	No of individuals	
			vgll3	six6				vgll3	six6
seabream_w1.1	Aegean Sea (Thermaikos Gulf)	2004	5 (4)	4 (4)	seabass_w1.1	Aegean Sea (Thermaikos Gulf)	2004	5 (5)	5 (5)
seabream_w1.2	Ionian Sea (Lagoon Klisova)	2004	5 (5)	5 (5)	seabass_w1.2	Ionian Sea (Lagoon Mesologgi)	2004	5 (5)	5 (5)
seabream_w1.3	Aegean Sea (Thracian Sea)	2005	5 (3)	3 (3)	seabass_w1.3	Ionian Sea (Sagiada)	2004	5 (5)	5 (5)
seabream_w1.4	Aegean Sea (Toronean Gulf)	2005	5 (5)	5 (5)	seabass_w1.4	Aegean Sea (Thermaikos Gulf)	2005	5 (4)	4 (4)
seabream_w1.5	Ionian Sea (Lagoon Mesologgi)	2005	5 (5)	5 (5)	seabass_w1.5	Aegean Sea (Thracian Sea)	2005	5 (5)	5 (5)
seabream_w1.6	Ionian Sea (Unknown)	2006	5 (3)	3 (3)	seabass_w2.1	Ionian Sea (Lagoon Mesologgi)	2013	4 (4)	4 (4)
seabream_w2.1	Aegean Sea (Thermaikos Gulf)	2013	5 (5)	5 (5)	seabass_w2.2	Ionian Sea (Unknown)	2013	5 (5)	5 (5)
seabream_w2.2	Aegean Sea (Thracian Sea)	2013	5 (5)	5 (5)	seabass_w2.3	Aegean Sea (Thermaikos Gulf)	2013	4 (4)	4 (4)
seabream_w2.3	Ionian Sea (Lagoon Mesologgi)	2013	5 (5)	5 (5)	seabass_w2.4	Aegean Sea (Thracian Sea)	2014	5 (5)	5 (5)
seabream_w2.4	Ionian Sea (Unknown)	2014	4 (4)	4 (4)	seabass_w2.5	Aegean Sea (Thermaikos Gulf)	2016	3 (2)	2 (2)
seabream_w2.5	Aegean Sea (Thermaikos Gulf)	2016	5 (1)	1 (1)	seabass_w2.6	Ionian Sea (Preveza)	2016	6 (2)	2 (2)
Total wild samples			54	45	Total wild samples			53	46
seabream_f1.1	Hatchery 1	2004	4 (4)	4 (4)	seabass_f1.1	Hatchery 7	2004	9 (7)	7 (7)
seabream_f1.2	Hatchery 1	2005	4 (4)	4 (4)	seabass_f1.2	Hatchery 8	2004	9 (9)	11 (9)
seabream_f1.3	Hatchery 2	2005	3 (2)	2 (2)	seabass_f2.1	Hatchery 9	2014	5 (5)	5 (5)
seabream_f1.4	Hatchery 3	2007	5 (5)	8 (5)	seabass_f2.2	Hatchery 10	2014	4 (4)	5 (4)
seabream_f2.1	Hatchery 3	2014	5 (5)	5 (5)	seabass_f2.3	Hatchery 11	2014	4 (4)	5 (4)
seabream_f2.2	Hatchery 3	2016	2	–	seabass_f2.4	Hatchery 12	2014	3 (3)	4 (4)
seabream_f2.3	Hatchery 4	2014	4 (3)	3 (3)	seabass_f2.5	Hatchery 12	2016	4 (4)	4 (4)
seabream_f2.4	Hatchery 5	2014	5 (4)	4 (4)	Total farmed samples			38	41
seabream_f2.5	Hatchery 6	2014	5 (4)	4 (4)					
Total farmed samples			37	34					

*Note*: The year of sampling, origin (specifically whether from Aegean or Ionian Sea) and the location of samplings are reported. Hatchery origin is not reported as it may be misleading due to exchange of breeders between hatcheries. In brackets, the number of individuals analyzed for both genes is reported.

#### 2.1.2 Primer design

To design primers for amplifying the gene regions of *vgll3* and *six6* in search for SNPs in the two studied fish species, we used the Primer3 program (Kõressaar et al., 2018; Untergasser et al., 2012) accessible through the Primer3web portal (version 4.1.0; last accessed 10‐Apr‐2023). We obtained the reference sequences for *vgll3* and *six6* for each species from GenBank. For gilthead seabream, we retrieved gene locations using the annotations associated with the fSpaAur1.1 genome assembly (GenBank accession: GCA_900880675.1; Chr9:24,909,923–24,912,747 for *vgll3* and Chr16:14,404,952–14,408,067 for *six6*) and obtained the intron/exon information from the associated gff file (GCF_900880675.1_fSpaAur1.1_genomic.gff). For European seabass, we obtained gene locations and intron/exon information from the annotations deposited at Ensembl for the European seabass genes (dlabrax2021, GenBank accession: GCF_905237075.1). We then mapped the gene sequences to the chromosome‐level assembly of the species, namely the European seabass_V1.0 assembly (GenBank accession: GCA_000689215.1), using blastn sequence similarity searches. To ensure evolutionary conservation of the binding site, we anchored all primers to exons. After designing the primer pairs, we tested them for successful PCR amplification and then Sanger‐sequenced the amplicons in subsets of farmed and wild‐collected samples from different origins to increase the likelihood of finding polymorphic SNPs in a cost‐efficient manner (Table 2). Qualified SNPs were investigated in all collected samples (Table 1).

**TABLE 2:** Primer sequence, amplicon size, genomic coordinates (assemblies *Sp. aurata*: GCA_900880675.2; *D. labrax*: GCA_000689215.1) and annealing temperature for each studied genomic region.

Species	Gene	Genomic coordinates	Annealing temperature (°C)	Primer sequence (5′‐3′)	Amplicon size (bp)
*Dicentrarchus labrax*	*vgll3*	9,203,305–9,203,951^a^	63	TACCCTCCCCGATACCTGG	646
				TGTGTGGACAGTGCAGGAC	
		9,202,574–9,203,170	61	TCTCTCCCTGTCTCCTCCTC	596
				CTGGGATGGATAGGTGCTGT	
		9,201,705–9,202,720	63	TGCCTGGATGTGATGTACCA	1015
				CTGGTGTCGCAGGTCCCT	
		9,201,381–9,201,802	61	GGAGCGCTCTGAAAACTTGT	421
				TGTTGTTGCTGGTGATGGTG	
	*six6*	11,589,507–11,590,303	61	TCTTTCTTTAACACCGCCCG	796
				ACACCGACTCGTTCTTGTTG	
		11,590,632–11,591,488^a^	63	GGCTACAGGACTTACACCCA	856
				AAGTACCACAGCAAGATCGC	
		11,588,596–11,589,739	61	GCTATCTATCCGCCCTCTTATTT	1149
				CCCTCCGATGATTTCCTATTGG	
		11,592,056–11,592,572	61	GAATGTGACATCTGACGGGC	515
				CGAAGAGACAGAGCAGATCG	
*Sparus aurata*	*vgll3*	24,911,798–24,912,208^a^	63	AACGTCTATCACCCTCACCC	410
				ACCAAACTGACGTCTTTGCT	
		24,910,950–24,911,675	63	GCTCCCATTCTGCTCCCA	725
				CCGGGTGGTTGAAGGAGAC	
		24,910,316–24,911,343	63	GAGCGCACTACCTCCCTG	1027
				CACCACGTCGCCAATGTC	
		24,909,934–24,910,621	61	CCAATCACAAGCGCTCTGAA	687
				CCGACTTTTAGCGCATGGTT	
	*six6*	14,404,868–14,405,650	61	GTCTTTCTTTAACAGCGTGCG	782
				GCACCGACTCGTTCTTGTTG	
		14,406,022–14,406,799^a^	63	AACCGCAGACAAAGAGACAG	777
				ACCCCTTATTAAACAACAAGCAC	
		14,403,964–14,405,087	63	GTGTGTCTGATACTAAAGCCGA	1123
				TCTATTGGACTTGGCAGATTGG	
		14,407,370–14,407,840	63	GTCATAGTGCCTGCGTTGAG	470
				CGAAGAGACAGAGCAGATCG	

^a^ Containing polymorphic SNPs.

#### 2.1.3 Sanger sequencing and SNP genotyping

To perform PCR, we used a total reaction volume of 25 μL, consisting of 100 ng of genomic DNA as the template, 0.05 units of Qiagen Taq polymerase, 2 mM dNTPs, 0.25 μL of each primer (100 μΜ) and 2.5 μL of 10x Reaction Buffer (Qiagen, Hilden Germany). We assessed the success of the PCR products by electrophoresis in 1.5% (w/v) agarose gels. We subjected the PCR products that amplified successfully to enzymatic cleanup using ExoI and rSAP (New England Biolabs) as per the manufacturer's protocol and outsourced the cleaned PCR products to the Genewiz company (Leipzig, Germany) for Sanger sequencing. To identify SNPs, we aligned the resulting sequences with the respective reference sequence of GenBank using the Geneious program (v.10.2.6; https://www.geneious.com; last accessed 10‐Apr‐2023). We further characterized the coding SNPs as missense or synonymous using the AliView program (v.1.28; Larsson, 2014).

#### 2.1.4 Statistical analysis of genotype frequency differences for domestication

We employed the non‐parametric Wilcoxon test to assess the statistical significance of the genotype frequency differences between farmed and wild populations. We also evaluated whether the observed genotype frequencies adhere to the Hardy–Weinberg equilibrium for farmed and wild populations for each SNP. To examine the influence of domestication and sampling time on our findings, we employed generalized linear models (GLMs) implemented with the *glm* function in R. Given that approximately half of the samples were collected at a later period, between 2012 and 2016 (gilthead seabream: *n* = 45 for *vgll3* and *n* = 36 for *six6*; European seabass: *n* = 48 for *vgll3* and *n* = 45 for *six6*), and the other half at an earlier period, between 2004 and 2007 (gilthead seabream: *n* = 46 for *vgll3* and *n* = 43 for *six6*; European seabass: *n* = 43 for *vgll3* and *n* = 42 for *six6*) (Table 1), we employed a new binary variable, the collection period of samples (2004–2007 and 2012–2016). This involved creating a new binary variable using the *cbind* function in R, with the most frequent allele or genotype level in the farmed populations as a reference level. Furthermore, we utilized a stepwise selection procedure with Akaike information criterion (AIC), incorporating the ‘forward/backward’ strategy in R to identify the model that best fitted the data given the factors of ‘origin’ (farmed and wild populations) and ‘time’ (two periods of collection: 2004–2007 and 2012–2016) as described above (*stepwise* function in R; stepAIC(origin * time)). To account for the proportions of the data, we used a binomial error with the logit function. The AIC is defined as −2*log‐likelihood + 2*npar, where *likelihood* is the likelihood corresponding in each model and *npar* represents the number of parameters in the fitted model. All statistical analyses and visualizations were conducted in R v.3.6.1 (R Core Team, 2021).

To address the confounding factor of known marked reductions in effective population sizes in the farmed populations due to breeding programs, we simulated bottleneck scenarios with parameters matching the described characteristics of the farmed populations in the two species. Specifically, we developed custom Python code to simulate bottlenecks, reducing the effective population size from 10^6^ in gilthead seabream and 10^4^ in European seabass to 100 individuals. These reductions are consistent with historical events documented to have occurred about 5–10 generations ago (Saura et al., 2021). To conduct the simulations, we utilized observed *vgll3* and *six6* genotype frequencies from the wild populations, assuming a wild origin of the farmed populations. We ran 1000 simulations to examine the effects of the bottleneck on changes in these frequencies and employed a Fisher exact test to assess the significance of these changes in each permutation. In these simulations, we assumed a negligible impact of mutation on the two evolutionarily conserved candidate genes (script available at the GitHub link provided in the Data Availability section).

### 2.2 Chromosome‐level genotyping

We utilized whole‐genome sequencing data of pooled samples (Pool‐Seq) from 14 farmed and 10 wild populations of gilthead seabream, as well as from 12 farmed and 12 wild populations of European seabass. These populations originated from nine countries within the Mediterranean Sea region (Peñaloza et al., 2021; Table 3). The Illumina reads used in this study were previously employed to develop a SNP chip for these two fish species, facilitating streamlined population genomic analyses (Peñaloza et al., 2021). In this study, we meta‐analysed this data, which consisted of 31,989 million total reads from 93 HiSeq runs. Our aim was to investigate the impact of domestication on the genetic variation of the chromosomes containing the *vgll3* and *six6* genes in each species. Specifically, we focused on chromosomes 9 and 16 harbouring the *vgll3* and *six6* genes in gilthead seabream, as well as on linkage groups 15 and 12 containing the *vgll3* and *six6* genes in European seabass.

**TABLE 3:** Information about the country and the farmed or wild origin of the studied Pool‐Seq samples (adapted from Peñaloza et al., 2021).

Gilthead seabream (*Sparus aurata*)					European seabass (*Dicentrarchus labrax*)				
Origin	Population ID	Country	Number of individuals per pool	Number pools prepared	Origin	Population ID	Country	Number of individuals per pool	Number pools prepared
Farmed	Seabream_f1	France	25	2	Farmed	Seabass_f1	France	12	1
	Seabream_f2	Spain	25	2		Seabass_f2	Spain	25	2
	Seabream_f3	Spain	25	2		Seabass_f3	Spain	25	2
	Seabream_f4	Italy	25	1		Seabass_f4	Italy	25	2
	Seabream_f5	Croatia	25	2		Seabass_f5	Croatia	25	2
	Seabream_f6	Greece	14	1		Seabass_f6	Croatia	25	2
	Seabream_f7	Greece	13	1		Seabass_f7	Greece	25	2
	Seabream_f8	Greece	25	2		Seabass_f8	Greece	25	2
	Seabream_f9	Greece	25	2		Seabass_f9	Greece	25	2
	Seabream_f10	Greece	25	2		Seabass_f10	Greece	25	2
	Seabream_f11	Israel	25	2		Seabass_f11	Greece	25	2
	Seabream_f12	Egypt	15	1		Seabass_f12	Greece	25	1
Wild	Seabream_w1	Spain	25	2		Seabass_f13	Cyprus	25	2
	Seabream_w2	Spain	25	2		Seabass_f14	Egypt	15	1
	Seabream_w3	Tunisia	25	2	Wild	Seabass_w1	France	25	2
	Seabream_w4	Italy	25	2		Seabass_w2	Spain	11	1
	Seabream_w5	Italy	25	2		Seabass_w3	Morocco	25	2
	Seabream_w6	Greece	25	2		Seabass_w4	Italy	25	2
	Seabream_w7	Greece	25	2		Seabass_w5	Croatia	12	1
	Seabream_w8	Greece	25	2		Seabass_w6	Greece	25	2
	Seabream_w9	Greece	25	2		Seabass_w7	Greece	25	2
	Seabream_w10	Greece	25	2		Seabass_w8	Cyprus	15	1
	Seabream_w11	Turkey	25	2		Seabass_w9	Turkey	25	2
	Seabream_w12	Turkey	25	2		Seabass_w10	Turkey	25	2

#### 2.2.1 Read mapping

We obtained the Pool‐Seq data for each population from the NCBI Sequence Read Archive under the accession ID PRJEB40423. To ensure data quality, we filtered the sequences using the Trimmomatic tool (v. 0.38, Bolger et al., 2014) with the following parameters (paired‐end mode): ILLUMINACLIP: TruSeq3‐PE.fa:2:30:10; LEADING:5; TRAILING:5; SLIDINGWINDOW:3:15; MINLEN:100. Subsequently, we mapped the filtered reads to the reference assembly of each species (*S. aurata*: GCA_900880675.2; *D. labrax*: GCA_000689215.1) using the bwa mem algorithm (Li & Durbin, 2009). Finally, we extracted only the properly paired reads with mapping quality at least 15 (corresponding to a maximum 3% misalignment probability) using samtools (v. 9.2.0, Li et al., 2009).

#### 2.2.2 SNP genotyping

To obtain accurate genotype frequencies, we processed the properly paired reads for each population in Table 3 by sorting and merging between technical replicates using samtools. Subsequently, we utilized the bam‐readcount v.1.0 tool (Khanna et al., 2022) to obtain read counts for each genomic position with mapped reads. This resulted in an average of 30,688,390 genotyped positions in gilthead seabream and 23,960,088 genotyped positions in European seabass. The disparity in genotyped positions between the species can be attributed to the use of a different sequencing platforms (HiSeq X Ten for gilthead seabream and HiSeq 4000 for European seabass). We applied an AWK script to filter these positions, allowing for a minimum read depth of 25 counts. This threshold was determined after computer simulations of 1 million resampling events from a pool of 25 samples. The simulations demonstrated that a read depth of 25 counts provided an acceptable representation of at least half of all possible genotypes within each population pool; at the lower 95% confidence limit at least 13 samples from the pool of 25 samples are expected to be drawn. Allele frequencies below 1% were excluded to account for potential sequencing errors and incorrect mappings. It is worth noting that SNPs with such minor allele frequencies are commonly filtered out prior to population genomic analyses (Linck & Battey, 2019). Finally, we employed an in‐house Python function to identify the biallelic SNPs and their corresponding genotypes in each species. The Python scripts used for the simulations and the typing of biallelic SNPs are available at the GitHub link provided in the Data Availability section.

#### 2.2.3 Domestication‐associated chromosomal regions

The allele frequencies between farmed and wild populations were compared using two programs: PoPoolation2 (Kofler et al., 2011) and BayPass v. 2.1 (Gautier, 2015). Both programs accommodate Pool‐Seq experimental designs. In‐house Python code was used to produce input files for these programs. PoPoolation2 was used to calculate pairwise *F*_ST_ (the proportion of the total genetic variance contained in a subpopulation relative to the total genetic variance), Fisher's exact test (the determination if there is a statistically significant association exists between two categorical variables) and the Cochran–Mantel–Haenszel (CMH) test (the determination if there is a significant association between two categorical variables by stratifying the data with respect to a third variable) between farmed and wild populations for each SNP.

BayPass was executed in Pool‐Seq mode with a burn‐in of 10,000 iterations (double the default value) and recorded 10,000 samples with thinning (i.e. the number of iterations between two recorded samples) set to the default value of 25. Consequently, the post‐burn‐in length of the MCMC chain was 250,000 iterations. Other parameters were maintained at their default values. BayPass was employed to calculate the *XtX* differentiation statistic between farmed and wild populations and determine its significance for each SNP. The *XtX* is an estimation similar to *F*_ST_, but is corrected for the scaled covariance of population allele frequencies, providing estimates that are less sensitive by outlier populations (Günther & Coop, 2013). *P*‐values were adjusted for multiple testing using the Benjamini–Hochberg method (Benjamini & Hochberg, 1995) implemented in the *stats* package in Python. For regions exhibiting significant differentiation, information on neighbouring genes within a 50 Kbp window on both sides was extracted. This was achieved using available annotations (*.gff3 files) from BioMart for each species (Sparus_aurata.fSpaAur1.1.108 and Dicentrarchus_labrax. seabass_V1.0.105).

In addition, the biallelic SNPs identified at the gene‐level approach were cross‐referenced with the Pool‐Seq data. The population frequencies of these SNPs were examined using a non‐parametric Wilcoxon test to assess the statistical significance of the genotype frequency differences between farmed and wild populations in the Pool‐Seq dataset.

## 3 RESULTS

### 3.1 Α. Gene‐level genotyping

We PCR‐amplified nearly the entire transcribed region of *vgll3* and *six6* in gilthead seabream and European seabass using 16 pairs of primers (four primer pairs per genes per species; Table 2). PCR amplicons regions encompassed 97.4% and 80.5% of the transcribed regions of *vgll3* and *six6* in gilthead seabream, and 92.1% and 80.5% of the transcribed regions of *vgll3* and *six6* in European seabass, respectively. In each species, we identified one exonic biallelic SNP in *vgll3*, and two intronic biallelic SNPs in *six6*. The genomic locations of the SNPs are SNP_vgll3_: 9:24911884, SNP_six6_: 16:14406442 (henceforth SNP1), SNP_six6_: 16:14406471 (henceforth SNP2) in gilthead seabream, and SNP_vgll3_: 15:9203633, SNP_six6_: 12:11591053 (henceforth SNP1), SNP_six6_: 12:11591093 (henceforth SNP2) in European seabass. The genomic locations are according to the GCA_900880675.2 and GCA_000689215.1 genome assemblies in gilthead seabream and European seabass respectively. The exonic SNP was synonymous in gilthead seabream, whereas it resulted in a missense mutation (Ser332Ala) in European seabass. These SNPs did not concern the same codon between the two species.

**FIGURE 1:**
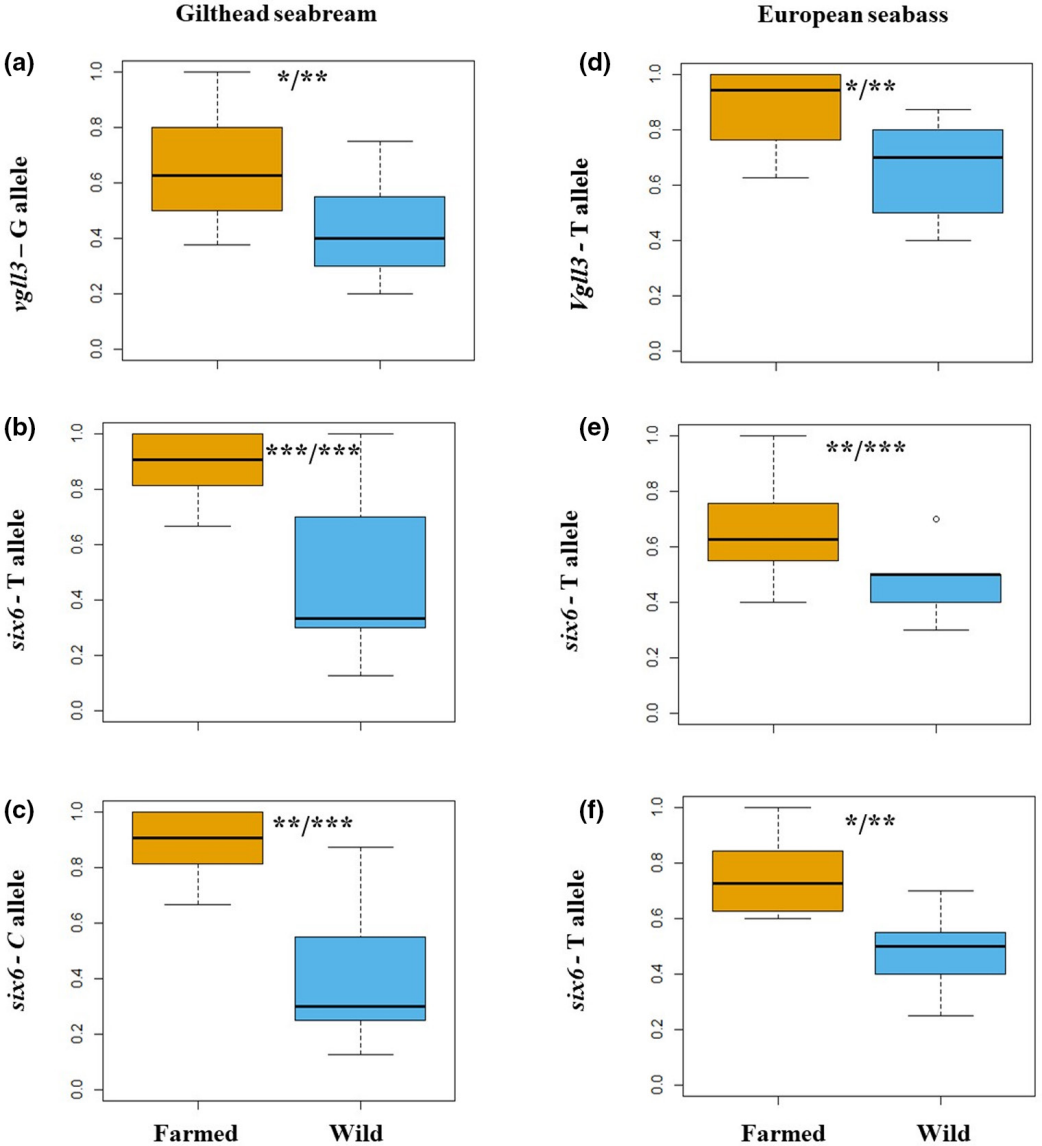
Boxplots illustrating the distribution of allele frequencies for the analysed SNPs in both farmed and wild populations of gilthead seabream (a–c) and European seabass (d–f). Asterisks denote significance levels between the sample groups (*p ≤ .05; **p ≤ .01; ***p ≤ .001). Left/right asterisks indicate the results from Wilcoxon test and GLM test respectively.

**FIGURE 2:**
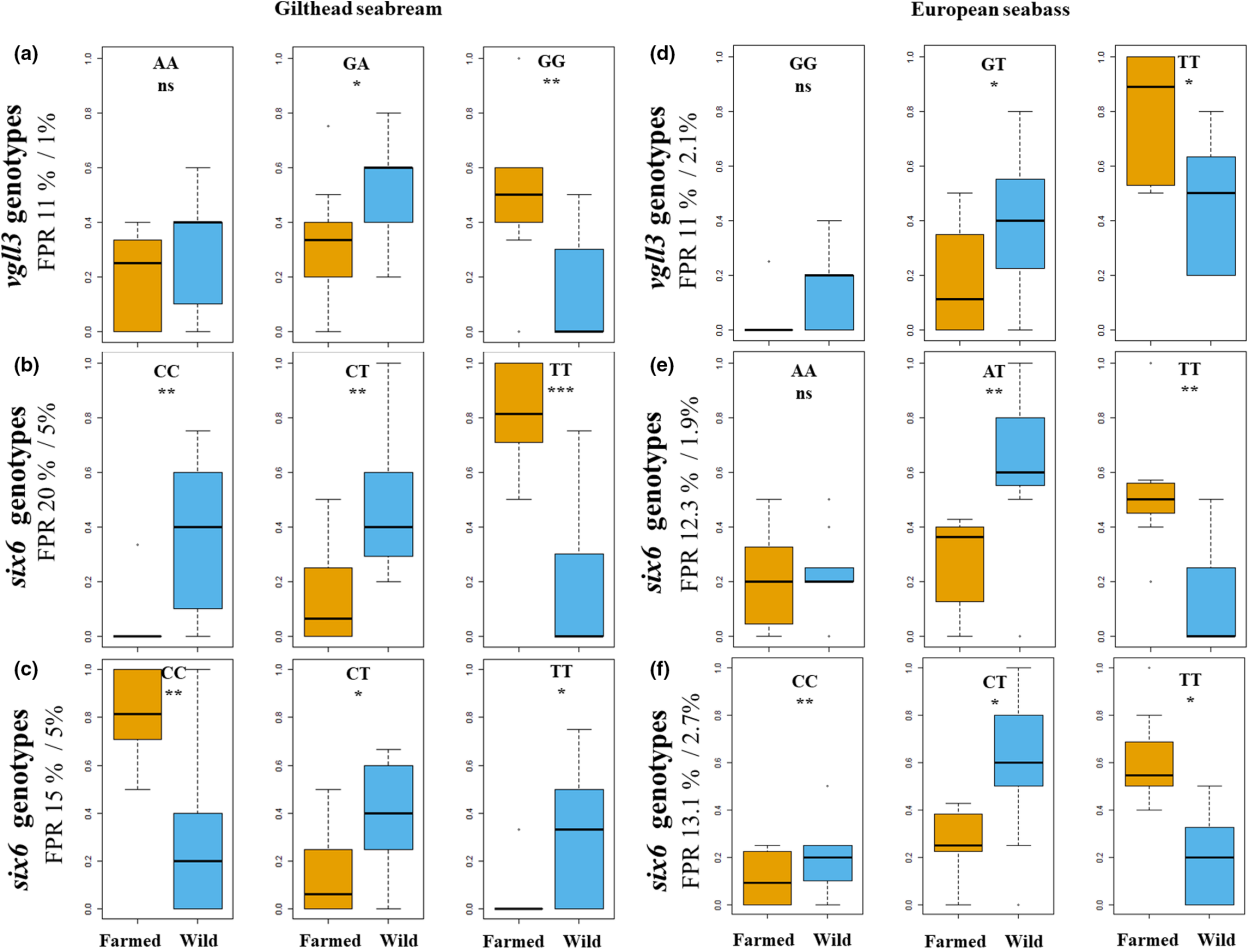
Boxplots illustrating the distribution of genotypes frequencies for the analysed SNPs in both farmed and wild populations of gilthead seabream (a–c) and European seabass (d–f). Asterisks denote significance levels between the sample groups (*p ≤ .05; **p ≤ .01; ***p ≤ .001). Left/right false‐positive rate (FPR) numbers on the y‐axis legends represent the percentage of permutations that were found significant at 5% and 1%, respectively, under the bottleneck scenario.

The genotype frequencies of all examined polymorphic SNPs showed significant differences between farmed and wild populations, as determined by Wilcoxon tests (gilthead seabream: *p* = 5.60e‐3 [*vgll3*]; *p* = 4.00e‐4 [*six6* – SNP1]; *p* = 3.70e‐3 [*six6* – SNP2]; European seabass: *p* = 3.95e‐2 [*vgll3*]; *p* = 3.50e‐3 [*six6* – SNP1]; *p* = 1.70e‐3 [*six6* – SNP2]; Figures 1 and 2). Wild populations exhibited higher heterozygosity compared to farmed populations (Figure 2), but both were found to be in Hardy–Weinberg equilibrium (wild populations: *p* = 5.61e‐2; farmed populations: *p* = 7.25e‐2). GLMs confirmed allele frequencies differed significantly between domesticated and wild populations on the datasets, especially for *six6* in gilthead seabream, even after accounting for time‐of‐sampling effects (gilthead seabream: *p* = 1.00e‐3 [*vgll3*]; *p* = 2.85e‐7 [*six6* – SNP1]; *p* = 2.50e‐8 [*six6* – SNP2]; European seabass: *p* = 3.80e‐3 [*vgll3*]; *p* = 1.30e‐3 [*six6* – SNP1]; *p* = 1.00e‐4 [*six6* – SNP2]; Figure 1). The time‐of‐sampling factor was found to be not significant, and the model with sample origin as the sole factor provided the best fit for the data based on AIC.

Our bottleneck simulations yielded a false‐positive rate (FPR) ranging from 11% to 20% in gilthead seabream, and from 11% to 13% in European seabass, at a 5% significance level. At a 1% significance level, FPR ranged from 1% to 3% for both species and the two candidate genes (Figure 2). In other words, even at a moderately relaxed 5% significance level, the likelihood of erroneously identifying significant differences in allele frequencies is well‐controlled in the face of bottlenecks in the farmed populations. Notably, the FPR significantly decreases when adopting a more stringent 1% significance level, reinforcing the robustness of the observed differences.

### 3.2 Chromosome‐level genotyping

We determined and analysed the genotype frequencies of 606,135 biallelic SNPs on chromosome 9 containing the *vgll3* gene (SNP density: 61.06 ± 278.09 bp), and 585,183 biallelic SNPs on chromosome 16 containing the *six6* gene (SNP density: 51.39 ± 192.53 bp) in gilthead seabream. Similarly, we analysed 343,064 biallelic SNPs on linkage group 15 containing the *vgll3* gene (SNP density: 74.52 ± 127.18 bp), and 310,807 biallelic SNPs on linkage group 12 containing the *six6* gene (SNP density: 74.75 ± 133.6 bp) in European seabass. The significant difference in the reported number of SNPs, approximately 600 K on each of the gilthead seabream chromosomes compared to approximately 300 K on each of the European seabass chromosomes, can be attributed to technical factors. The gilthead seabream samples were sequenced using the higher throughput HiSeq X Ten platform, whereas the European seabass pools were sequenced using the HiSeq 4000 platform (Peñaloza et al., 2021).

**FIGURE 3:**
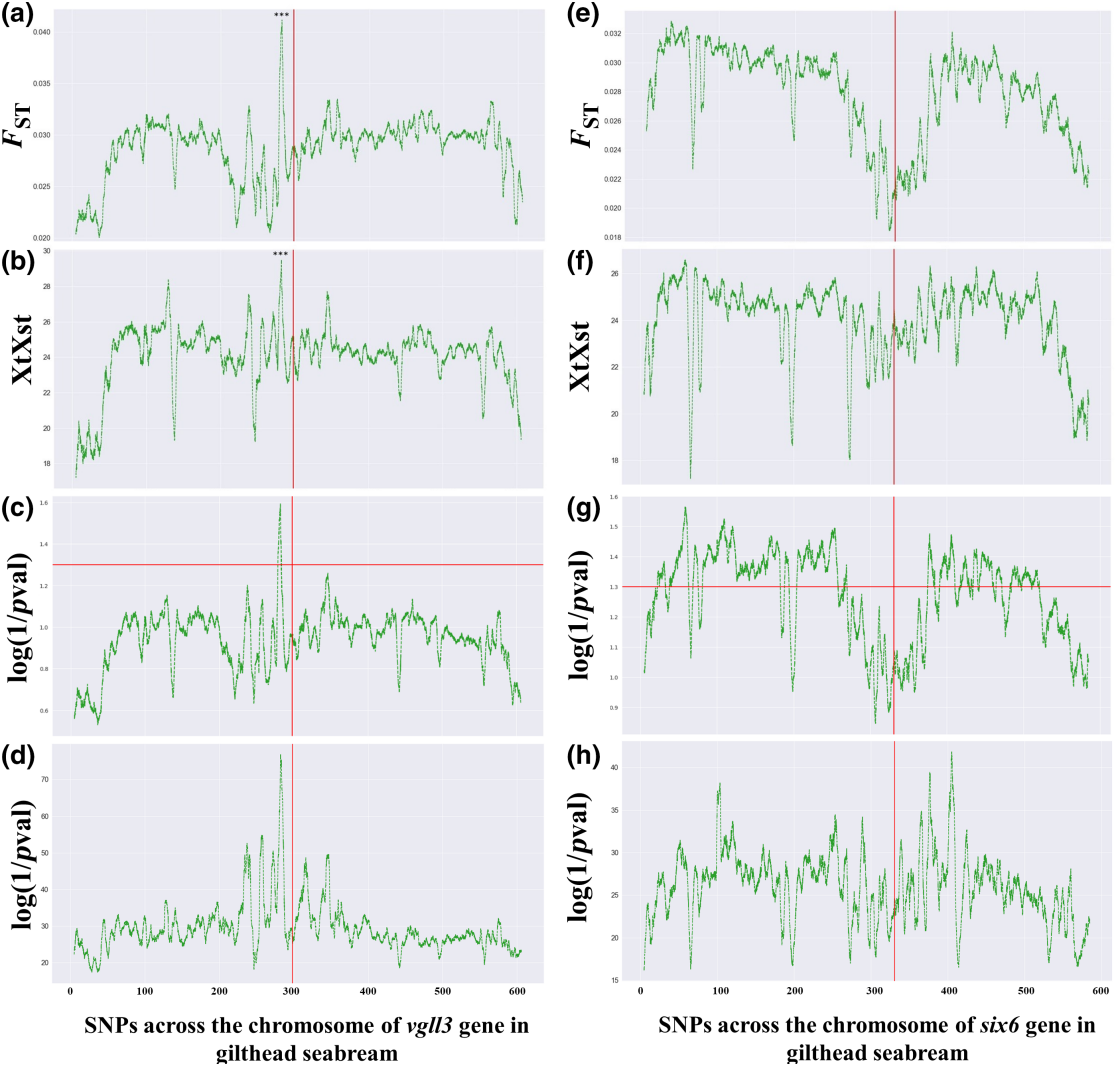
The landscape of genetic differentiation regarding domestication and statistical significance from Fisher's exact test and Cochran–Mantel–Haenszel test along the two studied chromosomes in gilthead seabream. Panels ‘a–d’ display the rolling average over 5000 SNPs of *F*_ST_, *XtX* values, Fisher's exact test and Cochran–Mantel–Haenszel test *p*‐values for chromosome 9, which encompasses the *vgll3* gene. Panels ‘e’ and ‘h’ exhibit the corresponding values for chromosome 16, which contains the *six6* gene. The vertical red lines indicate the position of the candidate genes in each case, while asterisks denote the level of corrected significance of the SNP with maximum *F*_ST_ and *XtX* values (***p ≤ .001). The horizontal red lines indicate the value of the log(1/*p*‐val) which corresponds to the *p* = .05. The values in *x*‐axis represent kilobases.

**FIGURE 4:**
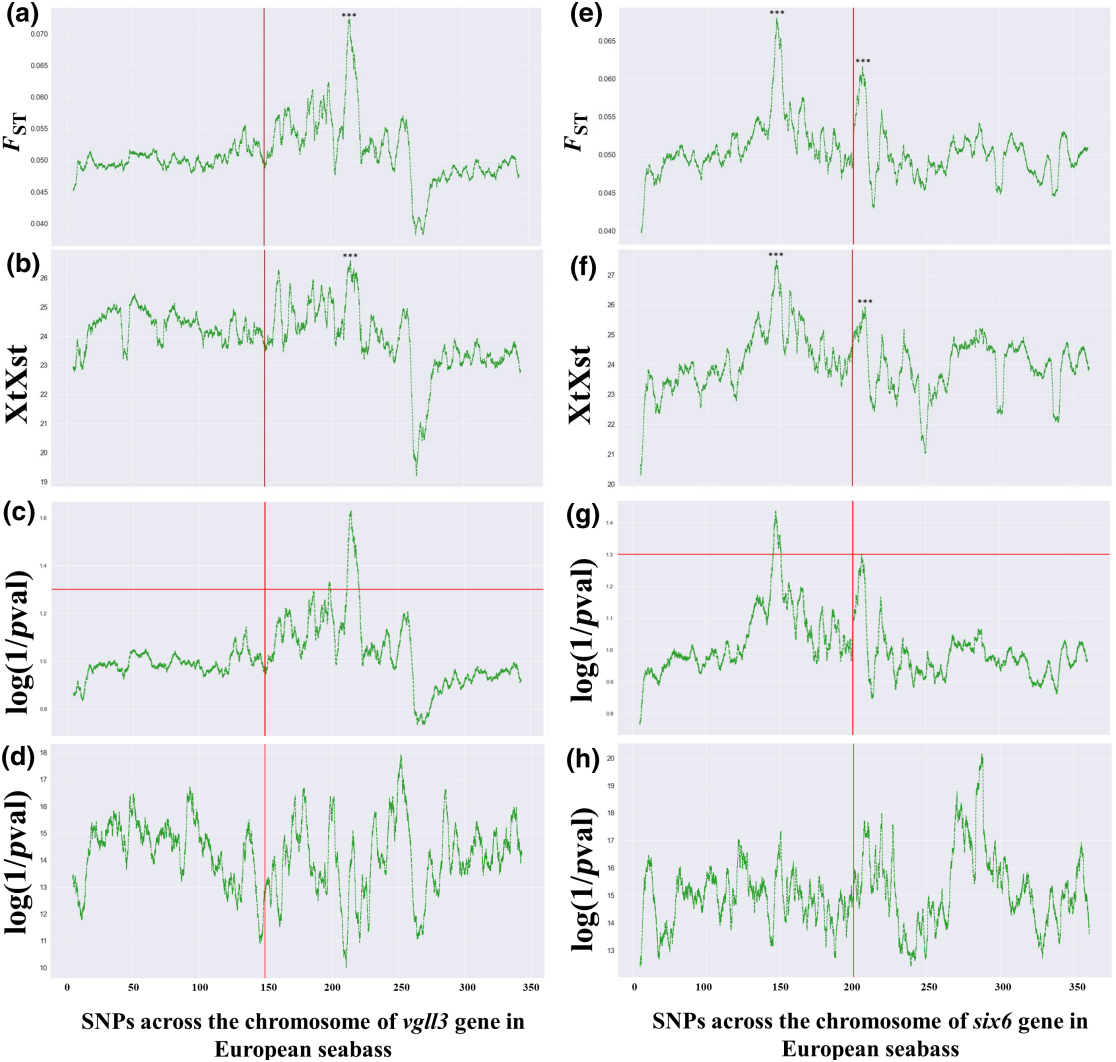
The landscape of genetic differentiation regarding domestication and statistical significance from Fisher's exact test and Cochran–Mantel–Haenszel test along the two studied chromosomes in European seabass. Panels ‘a–d’ display the rolling average over 5000 SNPs of *F*_ST_, *XtX* values, Fisher's exact test and Cochran–Mantel–Haenszel test *p*‐values for linkage group (LG) 15, which encompasses the *vgll3* gene. Panels ‘e’ and ‘h’ exhibit the corresponding values for LG12, which contains the *six6* gene. The vertical red lines indicate the position of the candidate genes in each case, while asterisks denote the level of corrected significance of the SNP with maximum *F*_ST_ and *XtX* values (***p ≤ .001). The horizontal red lines indicate the value of the log(1/*p*‐val), which corresponds to the *p* = .05. The values in *x*‐axis represent kilobases.

The chromosome‐wide estimates of genetic differentiation showed similar patterns to the results obtained from the gene‐level genotyping dataset. Notably, we identified a single highly differentiated region may be involved in the domestication process (*F*_ST_ = 0.133; *XtX* = 78.29; *p*_adj_ = 5.16e‐8; CMH = 1.942e‐189) located at 2.5 Mbp upstream of the *vgll3* gene in gilthead seabream (Figure 3a–d). Similarly, in European seabass, a single region was identified on the chromosome containing the *vgll3* gene, although it was located at 7 Mbp downstream (*F*_ST_ = 0.237; *XtX* = 63.85, *p*_adj_ = 3.48E‐07; CMH = 2.083e‐128; Figure 4a–d). Two additional regions associated with domestication were found in European seabass, one located at 0.7 Mbp downstream of the *six6* gene (*F*_ST_ = 0.218; *XtX* = 55.34; *p*_adj_ = 5.00e‐04; CMH = 4.62e‐213; Figure 4e–h) and the other located at 6 Mbp upstream (*F*_ST_ = 0.217; *XtX* = 58.84; *p*_adj_ = 2.00e‐05; CMH = 1.04e‐296; Figure 4e–h). However, we did not observe any highly differentiated regions in the chromosome containing the *six6* gene of gilthead seabream. Instead, we found a surprising region of very low relative differentiation with a length of about 105 kbps surrounding the exact location of the *six6* gene (Figure 3e,f). These results were consistent about the existence and position of differentiation regions in both species across the different statistical tests we performed, including *F*_ST_, *XtX*, Fisher's exact and CMH tests (Figures 3 and 4).

The biallelic nature of the SNPs of the gene‐level approach was confirmed in the Pool‐Seq data. The two studied SNPs of *six6* in gilthead seabream were further found to have a significant difference between farmed and wild populations in the Pool‐Seq data both in the PoPoolation2 and BayPass analyses (SNP1: *F*_ST_ = 0.092; *XtX* = 69.75; *p*_adj_ = 7.30e‐03; CMH = 1.79e‐183; SNP2: *F*_ST_ = 0.103; *XtX* = 71.08; *p*_adj_ = 5.20e‐03; CMH = 4.05e‐234) and with non‐parametric Wilcoxon tests (*six6* – SNP1: *p*_adj_ = 3.70e‐3; *six6* – SNP2: *p*_adj_ = 2.40e‐2). Overall, the Pool‐Seq data revealed 41 biallelic SNPs were found on the *vgll3* gene of gilthead seabream (average *F*_ST_ = 0.014; *XtX* = 20.71; *p*_adj_ = 0.733; CMH = 0.039), 12 SNPs on *six6* gene of gilthead seabream (average *F*_ST_ = 0.014; *XtX* = 20.71; *p*_adj_ = 0.733; CMH = 2.277), 34 SNPs on *vgll3* of European seabass (average *F*_ST_ = 0.041; *XtX* = 20.90; *p*_adj_ = 0.781; CMH = 0.127) and 21 SNPs on *six6* of European seabass (average *F*_ST_ = 0.053; *XtX* = 21.86; *p*_adj_ = 0.844; CMH = 0.034).

### 3.3 Novel candidate genes for domestication

Through the analysis of genes within 100 kbp of the identified peaks of differentiation, we discovered a range of potential regulatory elements and additional candidate genes (Table 4). Notably, the sole genomic feature at the peak next to the *vgll3* gene in gilthead seabream contained a CpG island. Table 4 provides comprehensive information on the identified genes in each case.

**TABLE 4:** Annotated genes and regulatory elements detected in gilthead seabream and European seabass for regions within 100 kbp of the peaks with the maximum differentiation in each case.

Species	Chromosome	Peak position	Annotated gene/region	Gene position (bp)
*Sparus aurata*	L537129.1 (*vgll3* chromosome)	22,460,410	CpG island	22,430,167–22,430,571
*Dicentrarchus labrax*	HG916832.1 (*vgll3* chromosome)	16,286,816	Retinitis pigmentosa GTPase regulator (*rpgr*)	16,239,969–16,244,529
			Ornithine carbamoyltransferase (*oct*)	16,245,284–16,249,612
			G‐protein‐coupled receptor 161 (*gpr161*)	16,252,778–16,258,234
			ddb1 and cul4‐associated factor 6 (*dcaf6*)	16,261,017–16,284,118
			Mitochondrial pyruvate carrier 2 (*mpc2*)	16,286,421–16,291,829
			Ribosomal protein L24 (*rpl24*)	16,293,485–16,297,194
			Centrosomal protein 97 (*cep97*)	16,298,565–16,302,322
			Neurexophilin and PC‐esterase domain family, member 3 (*nxpe3*)	16,304,395–16,307,184
			Malic enzyme 3, NADP (+)‐dependent, mitochondrial (*me3*)	16,313,335–16,324,581
			Immunoglobulin‐like domain containing receptor 1b (*ildr1*)	16,325,825–16,335,625
*Dicentrarchus labrax*	HG916829.1 (*six6* chromosome)	12,321,585	Membrane‐bound O‐acyltransferase domain containing 2a (*mboat2*)	12,277,343–12,318,144
			Kinase D‐interacting substrate 220a (*kidin220*)	12,326,653–12,365,899
			Inhibitor of DNA binding 2a (*id2*)	12,368,259–12,370,413

## 4 DISCUSSION

In this study, we investigated the genetic differentiation in the *vgll3* and *six6* genes between farmed and wild populations and their potential role in the domestication process of two commercially significant fish species, namely the gilthead seabream and the European seabass. Our findings support their potential involvement in their domestication process, and thus in growth, given that domestication in these fish is closely linked to growth‐related traits. To ensure the robustness of our conclusions, we employed a comprehensive approach that involved the genotyping of two distinct sample collections.

### 4.1 Gene‐level genotyping: a role for *vgll3* and *six6* in the domestication process

The targeted SNP genotyping of *vgll3* and *six6* genes revealed significant allele and genotype frequency differences between farmed and wild populations suggesting that they may be involved in the domestication process (Figures 1 and 2). These findings remained significant even after considering the potential confounding factor of time‐of‐sampling. Demographic processes may also play a significant role in shaping the genetic landscape of populations. In our investigation, these processes may have contributed to the observed allele frequency differences. Notably, Saura et al. (2021) documented a relatively small effective population size (less than 100 individuals) in both species within farms. They also reported a marked drop in effective population sizes for gilthead seabream and European seabass approximately 5–10 generations ago, possibly attributed to the influence of intensive breeding programs (Saura et al., 2021). By simulating such bottleneck scenarios, we identified FPR between 11% and 20% at a 5% significance level and between 1% and 5% at a 1% significance level. This indicates that the risk of falsely identifying significant differences remains relatively manageable, especially at the lower significance threshold. Furthermore, Saura et al. (2021) acknowledged that their effective population size estimates might have been slightly underestimated and thus our FPRs are conservative in this regard. Taken together, our significant allele frequency differences appear to hold good validity, as they do not seem to be influenced solely by such demographic events.

It is known that the domestication of the two species, gilthead seabream and European seabass, has started in the 1980s (Felip & Piferrer, 2018; Gkagkavouzis et al., 2019), and the first commercial breeding programs were reported approximately 20 years ago in Greece (Gkagkavouzis et al., 2021; Thorland et al., 2006). It is worth noting that growth and growth‐related traits are commonly targeted in breeding programs for these species (Chavanne et al., 2016). The latter suggests that the selection for enhanced growth and maturation may have influenced the genetic variation in these genes, leading to their possible association with the domestication process. Similar studies conducted on Atlantic salmon comparing domesticated and wild populations, have also reported strong association signals in regions encompassing *vgll3* and/or *six6* genes (Ayllon et al., 2015; Sinclair‐Waters et al., 2020), an outcome, however, that may be influenced by farming conditions such as the feeding regime (Ayllon et al., 2019; Besnier et al., 2023). Identifying causative SNPs in this dataset is impossible; however, it is worth noting that the studied SNP in the *vgll3* gene of European seabass concerned a non‐synonymous mutation of a hydrophilic amino acid (serine) to a hydrophobic one (alanine). More experiments need to be performed to clarify the degree, if any, to which *vgll3* and *six6* may directly influence growth and maturation in gilthead seabream and European seabass.

### 4.2 Chromosome‐level genotyping

The Pool‐Seq dataset not only confirmed the presence of SNPs identified through the gene‐level approach, but also provided additional support for the allele frequencies differed significantly between domesticated and wild populations on the *six6* gene in gilthead seabream. This added good confidence to our findings as the Pool‐Seq analysis involved an independent set of populations with much more diverse biogeographic distribution. It seems plausible therefore to consider the *six6* gene as important target of the domestication process at least in gilthead seabream, and these may serve as focal points for investigating the mechanisms underlying domestication and its species effects. This is a key outcome given that fish domestication may be influenced by several variable factors, including culturing conditions and origin of breeders, which perhaps contributed to the lack of significant findings in the SNPs of the two candidate genes of European seabass in the Pool‐Seq dataset and the *vgll3* of gilthead seabream.

It is also intriguing to note that the genomic landscape of divergence differed significantly in the region surrounding the *six6* gene in gilthead seabream compared to other regions. The low levels of divergence in this region (Figure 3e,f), present a unique pattern that requires further investigation to fully understand its underlying causes. One possibility may be the influence of escaped farmed individuals, which could contribute to the homogenization effect in this specific region. Similar phenomena have been documented in other studies, such as the escape of farmed salmon impacting wild populations (e.g. Bolstad et al., 2021). Another possibility may be the renewal of the hatcheries brood stocks with individuals from wild populations as reported for gilthead seabream and European seabass by Villanueva et al. (2022). Alternatively, the low divergence might be indicative of the presence of purifying selection. Purifying selection has been associated with the maintenance of genetic stability in certain genomic regions (Cvijović et al., 2018). The presence of genomic ‘valleys’ with reduced divergence has been observed in various contexts, as mentioned in other studies in which genomic valleys were suggested to slow down the divergence of genomes during speciation in different species (Hofer et al., 2012; Roesti et al., 2012; Sendell‐Price et al., 2020; Van Doren et al., 2017; Wang, Street, et al., 2016); however, this has rarely been suggested in the context of domestication. Further research is necessary to shed light on the underlying mechanisms and to explore this interesting phenomenon in the context of domestication.

By meta‐analysing the Pool‐Seq dataset, we identified additional regions that exhibited significantly high levels of divergence between farmed and wild populations. These regions were located at distances of 0.7 Mbp from the *six6* gene in European seabass, 2.5 Mbp from the *vgll3* gene in gilthead seabream and 7 Mbp from the *vgll3* gene in European seabass. Notably, even SNPs located around 2 Mbps away from a gene of interest can still have functional effects (Brodie et al., 2016), thus indicating the potential significance of genetic variants in these regions for the regulation and function of the candidate genes. Moreover, the maximum *F*_ST_ values observed in these regions were particularly high, reaching approximately 13% in gilthead seabream and 21%–23% in European seabass. These values surpassed the levels commonly reported in previous population studies based on neutral molecular markers related to these species, which range from 2.2% to 5.9% in gilthead seabream (Gkagkavouzis et al., 2021; Polovina et al., 2020; Žužul et al., 2019) and up to 12% in European seabass. Based on these uncommonly high *F*_ST_ values, we may advocate that substantial genetic differentiation exists in the identified regions suggesting perhaps a potential influence of domestication and maybe on the genetic composition of the studied candidate genes in the two fish species.

Furthermore, our analysis of annotations within a 100 kbp window around each peak of differentiation revealed interesting candidate genes and regulatory regions that could have contributed to the domestication process in the studied species. Particularly important is the finding that the maximum differentiation in the *vgll3* chromosome of gilthead seabream occurs near a CpG island. CpG islands are known to have regulatory functions in gene expression (Lim et al., 2019). The association of CpG islands with the regulation of growth‐determining genes (Moore et al., 2013) and the connection between different isoforms of *vgll3* expression and variation in maturation age in Atlantic salmon (Verta et al., 2020) emphasize the role of the regulation of gene expression in determining phenotype, especially during development. Investigating the effect of the CpG island on the regulation of *vgll3* in gilthead seabream should be a priority in future research on growth rates in this species.

In European seabass, the analysis of a 100 kbp window around the identified region of maximum differentiation on the *vgll3* chromosome revealed the presence of several interesting genes, including the G‐protein‐coupled receptor 161 (*gpr161*). The *gpr161* has been linked to developmental processes in various species, such as in zebrafish (Leung et al., 2008) and Chinese pigs (Zhu et al., 2017). Notably, both the *vgll3* and *gpr161* genes have also been identified as selection signatures in sheep populations (Zhao et al., 2016). Another gene found in this region is *rpgr*, which has been linked to the vision ability in domesticated chickens (Wang, Zhang, et al., 2016). Vision appears to be a feature affected by domestication in fishes, as evidenced by the smaller eye size observed in domesticated Atlantic salmon (Perry et al., 2021). Additionally, the *six6* gene involved in eye development in vertebrates (Pritchard et al., 2018) is also relevant in this context. Another two genes found in the region are the *rpl24* and *nxpe3‐*. The *rpl24* gene is linked to production traits in Atlantic salmon (Liu et al., 2017), while *nxpe3* is involved in the regulation of neural crest cells in the dogs, which determine coloration, morphology and behaviour (Wilkins et al., 2014). Another gene was identified in the same region that encodes for a malic enzyme (*me3*), which participates in the citric acid cycle. A locus of a malic enzyme (mMEP‐2*) located on the chromosome of *vgll3* in Atlantic salmon, has been shown to affect early maturation (Morán et al., 2023). Other cases were also found, such as the *oct* gene participating in the urea cycle (Monzani & Moraes, 2008), and the *dcaf6* gene, a component of a ligase‐ubiquitin complex. Two more genes were also detected, that is, *ildr1* (Tong et al., 2021) and *mpc2* (Zangari et al., 2020) related to important traits for aquaculture such as immune response and metabolism respectively.

Similarly, in the *six6* chromosome of the European seabass, three additional genes were identified, that is, *mboat2*, *kidins220* and *id2*. Interestingly, the *mboat2* gene, along with *six6* and *vgll3*, has been linked to sea age at maturity in Atlantic salmon (Sinclair‐Waters et al., 2022). The expression of the *kidins220* gene in Chinook salmon was influenced by the modification of gastrointestinal tract microbiota with the use of antibiotics and probiotics (Sadeghi et al., 2022). The *id2* gene, together with the *id1* in teleosts, plays a role in the control the early myogenesis and the phenotype of the muscle fibres (Rallière et al., 2004). When taken together, all the above‐mentioned findings as discussed herein contribute to the improved understanding of the potential roles of these genes in the domestication process and their impact on important biological processes and traits in fish species.

In conclusion, the results of the present study underscore the potential association of *vgll3* and *six6* genes, along with their broader genomic regions, with the domestication of European seabass and gilthead seabream, utilizing two distinct approaches and datasets. These findings, combined with the evolutionarily conserved functions of *vgll3* and *six6* genes, warrant more comprehensive investigations into their roles in the maturation and growth of these two teleost species. Further research, including genotype–phenotype association studies and gene expression analyses throughout development, is necessary to elucidate the impact of these maturation/growth‐related genes in gilthead seabream and European seabass.

## AUTHOR CONTRIBUTIONS

**Aristotelis Moulistanos:** Data curation (equal); formal analysis (equal); investigation (equal); methodology (equal); project administration (supporting); visualization (supporting); writing – original draft (equal); writing – review and editing (equal). **Theopisti Nikolaou:** Methodology (supporting); writing – review and editing (supporting). **Smaragda Sismanoglou:** Methodology (supporting); writing – review and editing (supporting). **Konstantinos Gkagkavouzis:** Methodology (supporting); project administration (equal); visualization (supporting); writing – review and editing (supporting). **Nikoleta Karaiskou:** Methodology (supporting); project administration (supporting); writing – review and editing (supporting). **Efthimia Antonopoulou:** Investigation (supporting); methodology (supporting); supervision (supporting); writing – review and editing (supporting). **Alexandros Triantafyllidis:** Conceptualization (supporting); funding acquisition (supporting); investigation (supporting); methodology (supporting); project administration (supporting); supervision (equal); writing – review and editing (supporting). **Spiros Papakostas:** Conceptualization (lead); data curation (lead); formal analysis (lead); funding acquisition (lead); investigation (equal); methodology (equal); project administration (equal); resources (lead); software (lead); supervision (equal); validation (equal); visualization (equal); writing – original draft (equal); writing – review and editing (lead).

### OPEN RESEARCH BADGES

This article has earned Open Data and Open Materials badges. Data and materials are available at NCBI's Sequence Read Archive (SRA) accession number PRJEB40423 and https://github.com/spirospapakostas/MedFish_domestication.

## Data Availability

Raw sequence reads are available in NCBI's Sequence Read Archive (SRA) under accession number PRJEB40423. All the scripts developed for the data analysis are available on GitHub: https://github.com/spirospapakostas/MedFish_domestication.
